# Chip-Based High-Dimensional Optical Neural Network

**DOI:** 10.1007/s40820-022-00957-8

**Published:** 2022-11-14

**Authors:** Xinyu Wang, Peng Xie, Bohan Chen, Xingcai Zhang

**Affiliations:** 1 School of Future Technology, University of Chinese Academy of Sciences, Beijing, 100049 China; 2 Department of Engineering Science, University of Oxford, Parks Road, Oxford, OX1 3PJ UK; 3 School of Engineering, Massachusetts Institute of Technology, Cambridge, MA 02139 USA; 4 John A. Paulson School of Engineering and Applied Sciences, Harvard University, Cambridge, MA 02138 USA

**Keywords:** Integrated optics, Optical neural network, High-dimension, Mach–Zehnder interferometer, Nonlinear activation function, Parallel high-capacity analog computing

## Abstract

Parallel multi-thread processing in advanced intelligent processors is the core to realize high-speed and high-capacity signal processing systems. Optical neural network (ONN) has the native advantages of high parallelization, large bandwidth, and low power consumption to meet the demand of big data. Here, we demonstrate the dual-layer ONN with Mach–Zehnder interferometer (MZI) network and nonlinear layer, while the nonlinear activation function is achieved by optical-electronic signal conversion. Two frequency components from the microcomb source carrying digit datasets are simultaneously imposed and intelligently recognized through the ONN. We successfully achieve the digit classification of different frequency components by demultiplexing the output signal and testing power distribution. Efficient parallelization feasibility with wavelength division multiplexing is demonstrated in our high-dimensional ONN. This work provides a high-performance architecture for future parallel high-capacity optical analog computing.

**Supplementary Information:**

The online version contains supplementary material available at 10.1007/s40820-022-00957-8.

## Highlights

- High-dimensional optical neural network is achieved by introducing an on-chip soliton microcomb source and wavelength division multiplexing technique.
- The programmable electro-optic nonlinear layer and optical meshes promote the implementation of a multi-layer optical neural network.
- Ultra-low coupling loss is realized between functional chips and fiber array, which is around 1 dB per facet.

**Supplementary Information:**

The online version contains supplementary material available at 10.1007/s40820-022-00957-8.

## Graphical abstract

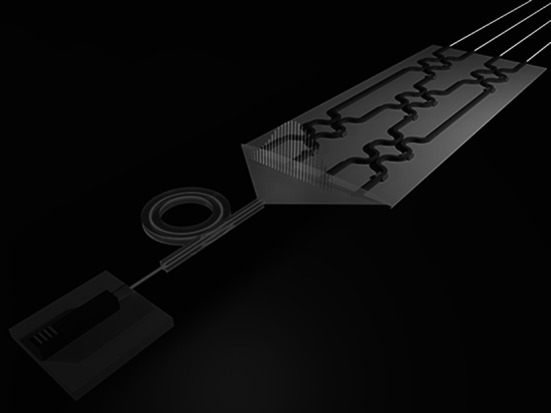

## Introduction

Deep neural network (DNN) has been an essential tool for developing general-purpose artificial intelligence (AI). The DNNs based on commercial electrical hardware processors or specifically optimized algorithms are extensively explored in pattern recognition, intelligent translation system, and material science [1–5]. With the rapid development of AI and increasing demand for high-capacity datasets processing, high-performance processors with accelerated matrix multiplication operations and high parallelism have attracted great attention in recent years. Progress of intelligent hardware plays a crucial role in developing next-generation advanced neural network processors. Nowadays, electronic neural network accelerators and processors based on the graphics processing unit (GPU), application-specific integrated circuits (ASIC), and field-programmable gate array (FPGA) dominate the commercial AI technique and specific function processing. However, intelligent electronic neural network hardware is still suffering from limited electrical bandwidth and huge energy consumption for the larger matrix decomposition. The computing carrier by electrons strictly restricts the computing capacity. It is worth mentioning that optical neural networks (ONN) based on photonic devices can compensate for the troublesome deficiencies of electrical hardware processors [6–9]. ONN can provide a higher speed with at least two orders of magnitude, lower power consumption, and larger bandwidth than conventional electrical artificial intelligent processors. To date, ONN has developed from free space optics [10–14] to integrated photonic devices [15–21]. Meanwhile, continuous innovations of nano-micro fabrication and photonic integrated devices pave the way for miniaturized intelligent photonics processers. Synchronous high-dimensional datasets processing or multi-thread operation is the urgent tendency with the arrival of the big data era. Photons have the natural superiorities of encoding high-dimensional information due to the abundant dimensions, such as polarization encoding [22–24], orbit angular momentum demodulation [25], optical frequency components encoding in communication, and quantum technology [26–29]. Photon takes the unique superiority of parallel information transmission and processing by introducing the wavelength division multiplexing (WDM) technique, which has been widely used in current high-capacity optical communication systems [30, 31] and parallel quantum key distribution [32]. Based on the independent propagation characteristic of photons, the rate of information throughput of ONNs depending on the bandwidth or quantities of available wavelengths can be increased exponentially. Assisted by the commercial WDM techniques, the soliton microcomb (SMC) source [33] has demonstrated that it could efficiently contribute to the implementation of high-speed and parallel photonic convolution image accelerators [34, 35]. Especially, SMC can also be employed for parallel different information processing in ONN systems, which is promising in rapid parallel scenario analysis or multi-thread information processing via utilizing the dimension of wavelengths, such as emotion recognition, gesture recognizer, Fourier transformation of signals, speech recognition, and computing accelerator.

In this letter, we propose an architecture of high-dimensional ONN, which consists of an on-chip SMC source, WDM module, and dual-layer ONN. In the dual-layer ONN structure, we introduce the chip-based Mach–Zehnder interferometer (MZI) network as the matrix multiplication linear layer and electro-optic nonlinear modules as the nonlinear activation function, which is composed of electro-optic modulators (EOM), detector modules, and FPGA control systems. The flexible programmable capability of the MZI network ensures multi-objection classification and recognition via learning from the different objection datasets. Integrated microresonator technology provides the automatic single SMC generation characterized by the coherent multi-wavelength light source with a frequency spacing of 49 GHz, which is compatible with the commercial WDM technique. Benefitting from the large width of photonics devices and WDM techniques, the parallel high-dimensional ONN processor based on chip-based single SMC is experimentally demonstrated via classifying the MNIST datasets. The recognition accuracies of the digit ‘0’, ‘2’, and ‘7’ from the MNIST datasets are around 85% for different wavelengths. This work paves the innovative routines for exploring the chip-based parallel high-capacity AI accelerator.

## Architecture of High-dimensional ONN and Experimental Results

### Architecture of Chip-based High-dimensional ONN

**Fig. 1:**
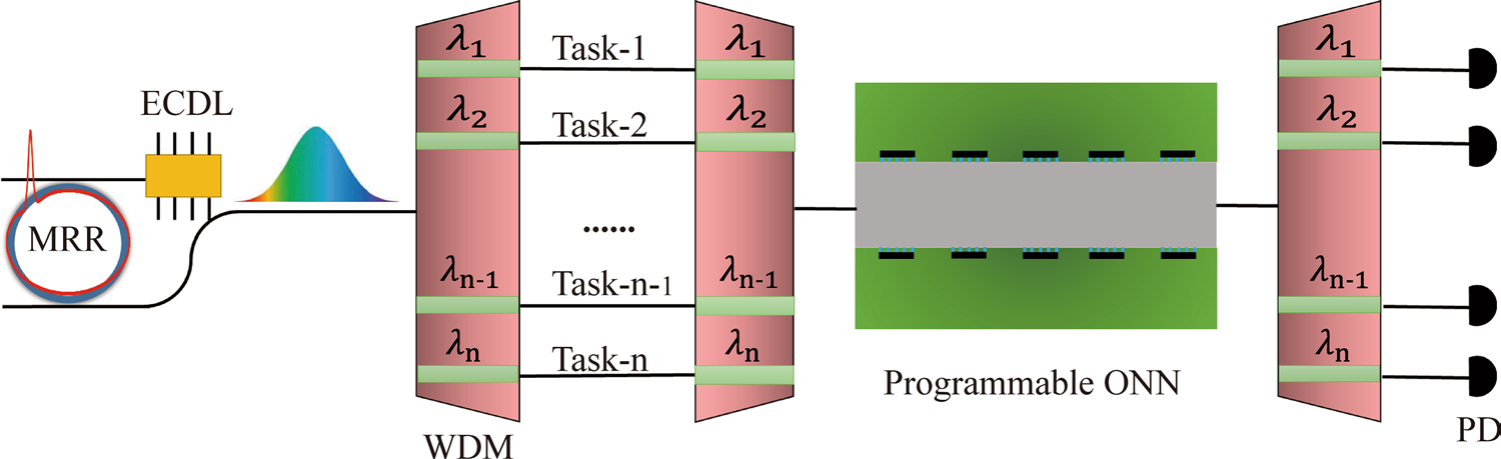
The concept illustration of chip-based high-dimensional ONN. The ONN technique supports parallel multi-signal processing, and multi-thread optical operations for multi-users by WDM technique. The narrow linewidth laser is the pumping source to generate the on-chip high repetition rate dissipative Kerr soliton source. The frequency components of the single SMC source are regarded as the multi-wavelength coherent lasers, which are used for realizing high-dimensional ONN via frequency multiplexing and demultiplexing. Each frequency component could be used for different recognition tasks or thread operations. MRR: Micro-ring resonator, ECDL: External cavity diode laser, WDM: Wavelength Division Multiplexing, PD: Photoelectric detector, ONN: Optical neural network

Figure 1 schematically illustrates the basic architecture of a high-dimensional ONN system based on SMC source and WDM techniques. The micro-ring resonator with high-quality factor is used to motivate the SMC source formation via sweeping the narrow linewidth laser into the red-detuned regime. Each frequency component via WDM could be regarded as one coherent laser and take one task, and then all components are coupled into the ONN chip to realize high-capacity signal processing and parallel high-dimensional computing. The flexible programmable characteristics of ONN guarantee the feasibility of different datasets processing and the demultiplexing technique is applied to divide the optical frequency components, respectively. The potential recognition information or computing results will be characterized by testing the power of output signals according to the classification results from the presupposed algorithm or optimization procedure.

### Single SMC Source with 49 GHz Repetition Rate

**Fig. 2:**
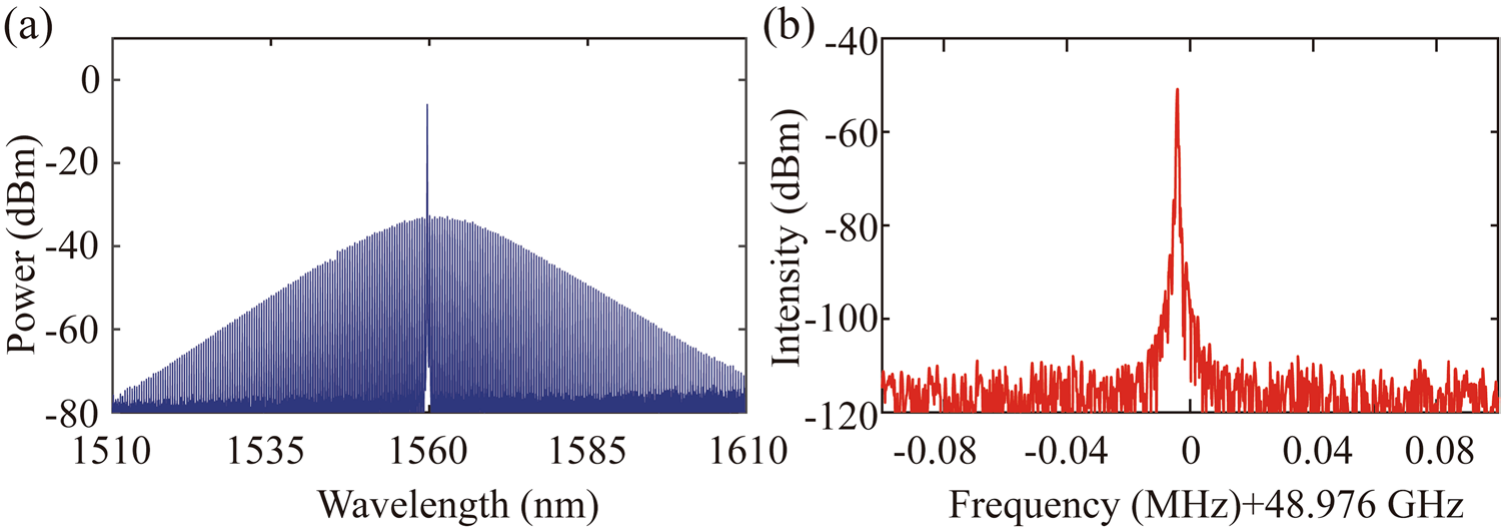
**a** The optical spectrum of the single SMC at the strong anomalous dispersion region. **b** The repetition rate of the single SMC source

Figure 2 shows the result of the single SMC generation. The basic principle of single SMC generation is based on the auxiliary laser heating the micro-cavity to reach the thermal balance. The auxiliary laser is shifted about 100 MHz from the pump laser via the acoustic optical modulator (AOM). The details of single SMC generation are demonstrated in the supplementary information. The proper frequency spacing between the pump and auxiliary laser could ensure the thermal balance in the soliton existence region or the red-detuned region. Once the pump laser is located at the soliton region, the external frequency modulation on the AOM and forward tuning of the pump laser frequency would make the microcomb state stabilize at the single SMC existence region. In our SMC source generation experiment, the micro-cavity is designed with strong anomalous dispersion and the pump and the auxiliary laser are located in the same resonance mode. The micro-cavity is fabricated at the platform of high-index doped silica glass with the radius of 592.1 μm. Pump laser is provided by the narrow linewidth laser at 1560.2 nm to be compatible with WDM. The generated optical spectrum of single SMC source is shown in Fig. 2(a), which covers the S, C, and L bands. As is shown in Fig. 2(b), the repetition rate is about 49 GHz, which meets the requirement of frequency spacing for avoiding crosstalk in dense WDM communication systems. By introducing the dense WDM technique, the single SMC source could be divided into many equidistant coherent lasers via demultiplexing to take several hundred tasks to realize parallel high-dimensional computing.

### Artificial Neural Network Based on Dual-layer ONN

**Fig. 3:**
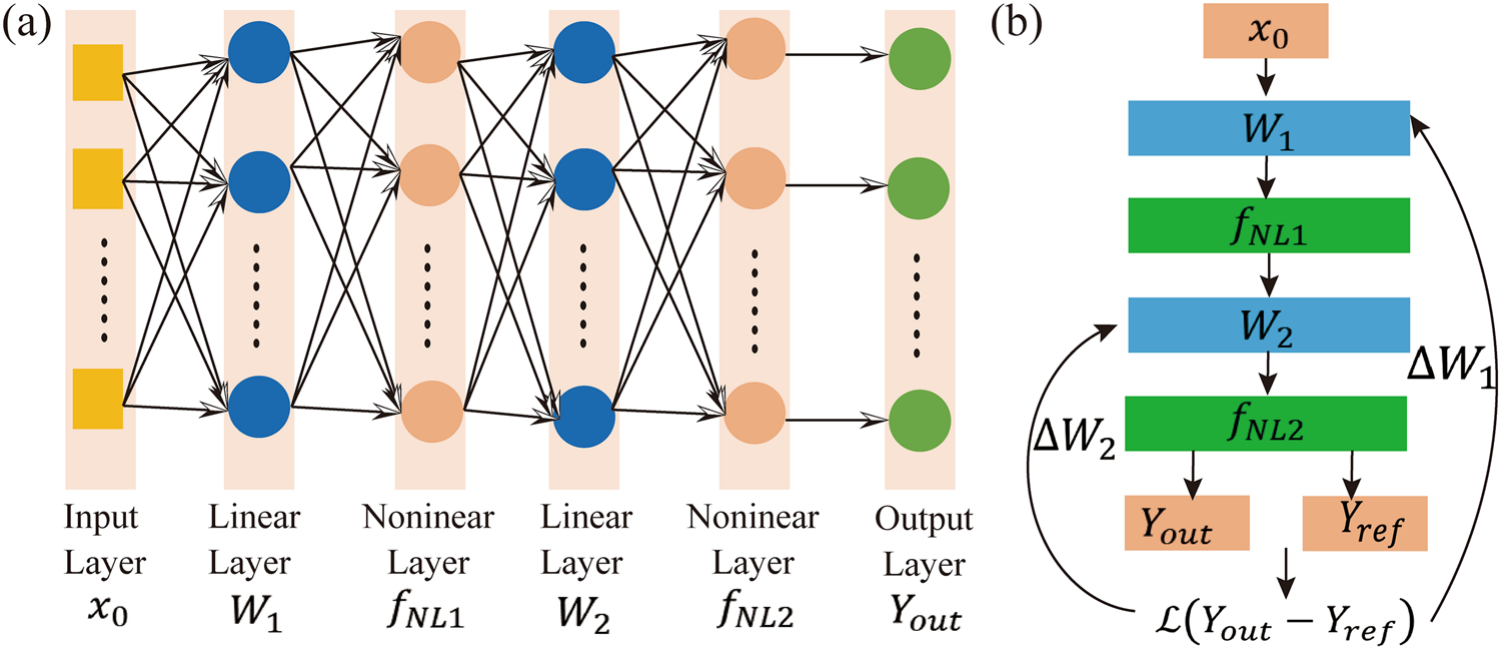
**a** Structure of the dual-layer ANN. **b** BP algorithm for optimizing the linear matrix. After completing the whole training process, the ANN could complete the specific task recognition. ANN: Artificial neural network

Figure 3(a) schematically illustrates the basic structure of artificial neural network (ANN), which is composed of an input layer (${x}_{0}$), cascade linear matrix multiplication layer (${W}_{1} \mathrm{and} {W}_{2}$) and nonlinear activation function layer (${f}_{NL1} \mathrm{and} {f}_{NL2}$), and an output layer (${Y}_{\mathrm{out}}$). Via loading the input data and matrix operations, the output vector ${Y}_{\mathrm{out}}$ is ${{f}_{NL2}(W}_{2} \times {{f}_{NL1}(W}_{1}{\times x}_{0}$)). For a given ANN, a lost function as the object error function is defined to minimize the target output and output prediction to adjust the matrix value. Such process is optimized via the back-propagating (BP) algorithm as shown in Fig. 3(b). The algorithm keeps adjusting the gradient of the matrix of ${W}_{1} \mathrm{and} {W}_{2}$ to minimize the objection function between experimental vectors and the target reference vectors value.

**Fig. 4:**
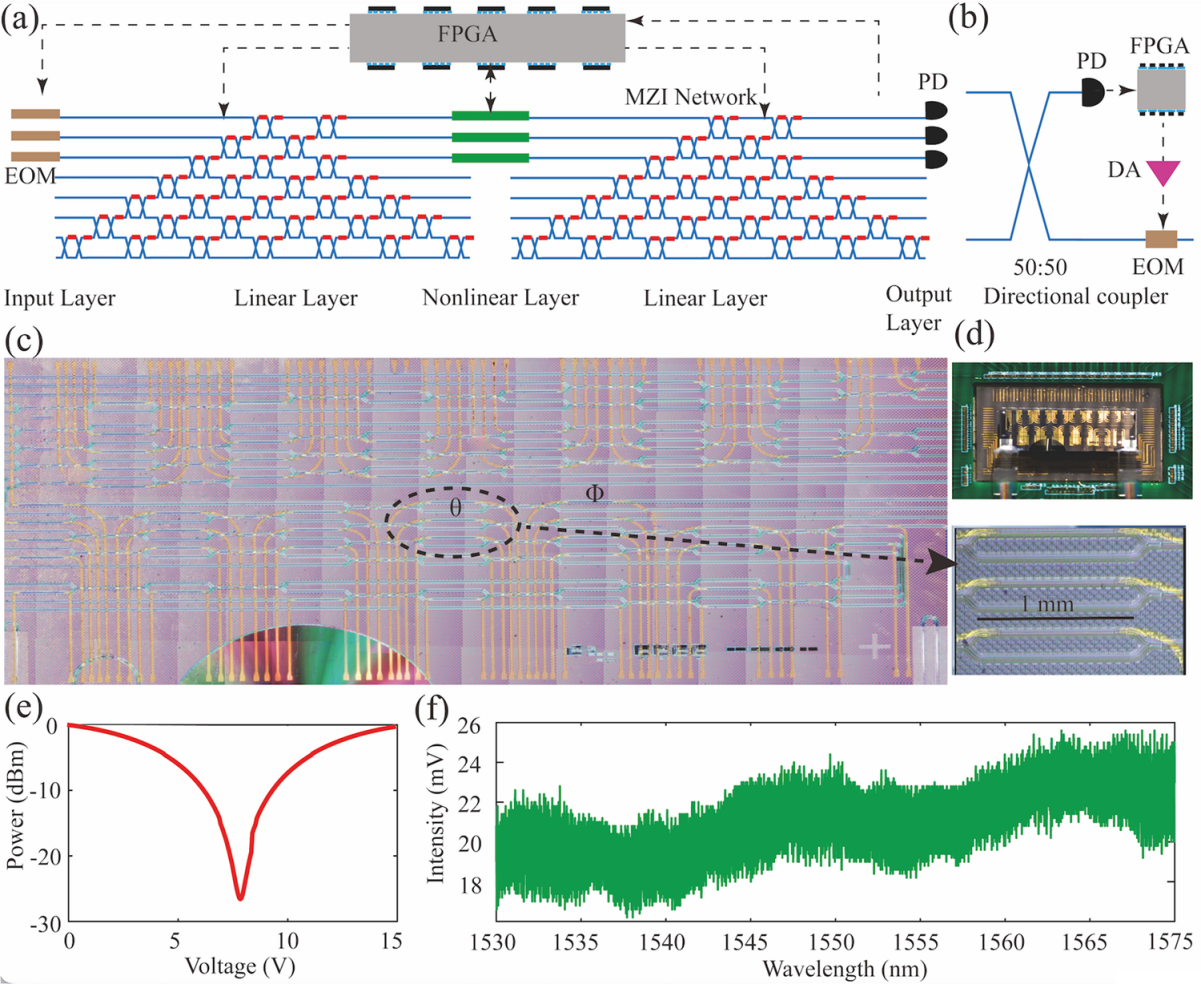
**a** Structure of dual-layer ONN. The dual-layer ONN is composed of electro-optic modulator as the input layer, MZI network as the linear layer, photoelectric conversion module as the nonlinear layer, and the PD as the output layer. **b** Schematic of the proposed nonlinear activation function which achieves a nonlinear response by converting a small portion of the optical input into an electrical signal, and then intensity modulating the remaining portion of the original optical signal as it passes through an interferometer. **c** A false-color micrograph of the MZI network with integrated heaters. The right picture is the local structure of MZI network. **d** Packaged chip. **e** Extinction of single MZI. **f** Transmission power trace when the input light experiences the four MZIs via sweeping the laser wavelength. EOM: electro-optic modulator, PD: photoelectric detector, DA: Digital to analog

Figure 4(a) shows the basic architecture of dual-layer ONN, which is composed of the MZI network as the linear matrix multiplication operation, and the photoelectric conversion module as the nonlinear layer. In the dual-layer ONN, light signals are encoded by the amplitude modulator. The linear layer is composed of the specific arranged MZIs fabricated in the platform of high-index doped silica glass. Each linear layer is composed of 29 programmable MZIs and each MZI corresponds to the neuron of ANN. The MZI network chip is packaged by the polarization-maintaining fiber array and thermally controlled via an external TEC controller as shown in Fig. 4(d). The coupling loss between the MZI network and polarization-maintaining fiber array is about 1 dB. The ultra-low coupling loss greatly contributes to the multi-layer ONN achievement. The transmission loss of the waveguide around 1560 nm is about 0.05 dB cm^−1^. Each MZI consists of phase shifter $\left(\theta \right)$ between two 50% directional couplers and an extra thermo-optic phase shifter $\left(\varnothing \right)$ as shown in Fig. 4(c). MZI implements the SU(2) transformation unitary matrix,

$$U_{{{\text{MZI}}}} = \frac{1}{2}\left( {\begin{array}{*{20}c} {e^{i\emptyset } \left( {e^{i\theta } - 1} \right)} & {ie^{i\emptyset } \left( {e^{i\theta } + 1} \right)} \\ {i\left( {e^{i\theta } + 1} \right)} & {1 - e^{i\theta } } \\ \end{array} } \right)$$

Matrices of the linear layer can be decomposed into sets of SU(2) transformation unitary matrix multiplication via the cascaded MZI. Via controlling external voltage to change the phase of the phase shifter $\left(\theta \right)$, the transmission matrix of the single MZI could be adjusted. Figure 4(e) shows the transmission trace via tuning the voltage on phase shifter $\left(\theta \right)$. According to the test results, 27 dB extinction ratio can be achieved, which means the directional coupler of beam splitter is fixed at 50:50. The high extinction ratio ensures arbitrary high-resolution matrix formation. Besides, the nonlinear activation function plays a critical role in ANNs by enabling them to learn complex mappings via minimizing the target vectors and reference vectors. Limited by the transformation efficiency, nonlinear motivation threshold power and flexible programming, the cascaded all-optical activation function is challengeable with the increasing layers of ONN. To overcome the challenge, we adopt an electro-optical architecture activation function to achieve the nonlinear layer computing. Figure 4(b) shows the basic structure of the nonlinear activation function. The light signal goes through the 50:50 directional coupler and is divided into two portions, one is transformed into the electrical signal via commercial PD, and another portion and be modulated by an amplitude modulator. The depth of ONN is determined by the efficiency of PD and loss of waveguide. The FPGA system or commercial computer could calculate the nonlinear function value via detected signal power and adjust the output of the amplitude modulator via an external controlled voltage source. And then the output optical signal would be modulated and then enter into the next linear matrix layer. By means of external FPGA and voltage source, the arbitrary nonlinear activation functions could be formed and XOR logic gate could be recognized by introducing the non-monotone nonlinear function in the single-layer ONN. The accuracy of nonlinear activation is determined by the extinction ratio of the amplitude modulator, which is about 23 dB in our experiment. In the dual-layer ONN, the second nonlinear layer and output layer are performed via the commercial PD and FPGA. Figure 4(f) shows the transmission power curve via sweeping the laser wavelength when the input light experiences the four MZIs. The transmission power curve is approximately identical, which proves that the matrix of MZI network has a relatively large bandwidth toward wavelength. The bandwidth of MZI is over 30 nm, which means that it can maintain similar matrix values and eigenvectors at different driving voltages at a large wavelength range. The large bandwidth of MZI network supports the implementation of synchronous processing of abundant datasets via multiwavelength encoding.

### Parallel Digit Classification via High-Dimensional ONN

**Fig. 5:**
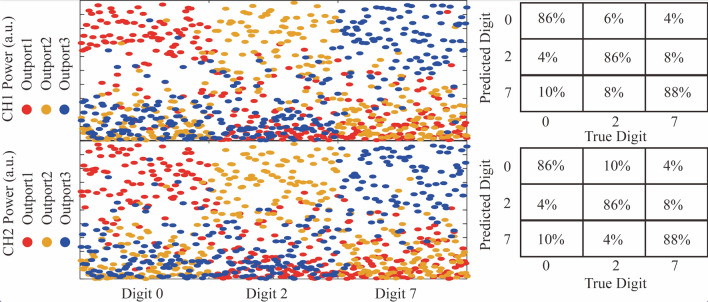
Experimental results of power distributions and recognition accuracies. The blue spots, red spots, and orange spots are the power ratios of output port1, output port2, and output port3, respectively. The accuracies from the two selected frequency components have been demonstrated, respectively

We evaluated the practicality of the high-dimensional dual-layer ONN by selecting two wavelengths (1562.6 nm and 1562.2 nm) of single SMC. The selected frequency components are amplified to 15 mW as the input signal and then sent to the same dual-layer ONN. The dense WDM is connected with the output ports of the second linear layer and the different wavelengths from the output signal are separated. By detecting the statistical power distribution of output ports from different wavelengths, the objected tasks could be recognized. In practice, we first choose the digit datasets from MNIST to train the dual-layer ONN. The digit of ‘0’,‘2’, and ‘7’ are selected to train in the dual-layer ONN system. Meanwhile, we choose the sigmoid function as the nonlinear activation function. Limited by the number of optical neurons, the original image couldn’t be directly loaded to ANN or ONN to accomplish the training process or power distribution statistics. The original image is divided into a series of pixel vectors, we sum up the total logarithm power of each port and statistic exponential power distribution after completing the training process. In the training process, the matrix parameters of dual-layer ONN can be obtained by using the standard back-propagation algorithm and the stochastic gradient descent method. The reference vectors of ‘0’,‘2’ and ‘7’ are, respectively, corresponding to (1 0 0), (0 1 0), and (0 0 1) for the output ports. The target recognition can be obtained by comparing the power distributions and object power vector values. Figure 5 illustrates the experimental recognition results based on the dual-layer ONN architecture. When the loaded digit datasets are imposed from ‘0’ to ‘7’, the output port of maximum power is changed from the first output port to the third output port via continuously loading the test digit datasets, which is consistent with the pre-established objection vector. Besides, the recognition accuracies for different wavelengths are approximately identical and the accuracies are about 85%. It should be mentioned that the programmable flexibility from MZI network and nonlinear control unit supports the multi objections recognition via learning from the different datasets. The large bandwidth of the MZI network and high-repetition-rate single SMC support the high-dimensional ONN to accomplish high-capacity computing.

## Discussion

The chip-based high-dimensional ONN combined with SMC source and WDM technique is preferred and superior in future-oriented big dataset recognition and parallel multi-thread signal processing. In this work, on-chip microcomb technology provides a pivotal multi-wavelength light source, which can carry different information via utilizing several frequency components to realize high-speed and high-capacity signal processing systems. The microcomb also has the native advantage to realize the high repetition rate or large frequency spacing by the careful geometric design, so that it could efficiently avoid signal crosstalk in parallel multi-thread processing. Moreover, the large bandwidth of the MZI network provides an opportunity to allow several wavelengths to maintain similar matrix values via a thermal-controlled MZI network and supports the different wavelengths with the same datasets to transmit in the same MZI network. The combination of a stable single SMC source, MZI networks, and WMD technique is an efficient approach to developing high-dimensional optical neural networks for high-cavity parallel recognition systems and signal processing. It is worth mentioning that our functional chips of SMC and MZI networks are based on the same material platform and compatible with CMOS technology. What is more, we achieve an efficient device package and ultra-low coupling loss. The details and methods of the device packaging are presented in the supplementary information. The challenge to the recognition accuracy is the cross-talk between different MZIs during the thermal tuning process. The possible solution is applying a faster adjuster, electro-optic modulation, or thermal deposition to reduce the thermal crosstalk. The integrated turn-key single SMC source [36–38], on-chip modulator [39, 40], amplifier [41], detector [42], WDM, low-loss waveguide [43, 44], optical storage [45], and excellent coupling will promote the implementation of the future-oriented monolithic multi-layer ONN processor and their applications.

## Conclusion

In summary, we propose a novel and powerful architecture of chip-based high-dimensional ONN based on dual-layer ONN, optical dissipative Kerr soliton microcomb source and WDM technique. We build the dual-layer ONN toward high-dimensional computing, which is composed of large bandwidth MZI networks and electro-optic modules. Based on the system, we successfully reach the goal of high-dimensional digit recognition by simultaneously loading the signals onto two different frequency components from the single SMC source. By demultiplexing the output signal and testing power distribution from the output ports of dual-layer ONN, the digit recognition accuracies of the two frequency components are about 85%. Besides, we achieve an efficient device package and ultra-low coupling loss between functional chips and fiber array, which is around 1 dB per facet. This work provides significant potential in high-capacity multi-dimensional datasets processing.

## Supplementary Information

Below is the link to the electronic supplementary material.

Supplementary file1 (DOCX 2824 kb)

## Data Availability

All data needed to evaluate the conclusions are present in the paper and/or the Supplementary Information.
