# Supplementary material for: Chip-Based High-Dimensional Optical Neural Network
Source: Nanomicro Lett. 2022 Nov 14;14:221. doi: 10.1007/s40820-022-00957-8 (PMC9663775; doi:10.1007/s40820-022-00957-8)
Supplement: Supplementary file 1 — Supplementary file1 (DOCX 2824 kb) [file 40820_2022_957_MOESM1_ESM.docx]

**Supplementary information for**

**Chip-based High-dimensional Optical Neural Network**

**Xinyu Wang^1,^** ^†^**, Peng Xie^2,^** ^†^**^,^** ***, Bohan Chen^2^, Xingcai Zhang^3,4,^***

^1^School of Future Technology, University of Chinese Academy of Sciences, Beijing 100049, China

^2^Department of Engineering Science, University of Oxford, Parks Road, Oxford OX1 3PJ, UK

^3^ School of Engineering, Massachusetts Institute of Technology, Cambridge, MA 02139, USA

^4^ John A. Paulson School of Engineering and Applied Sciences, Harvard University, Cambridge, MA 02138, USA

^†^These authors contribute equally.

*Corresponding email: [peng.xie@eng.ox.ac.uk](mailto:peng.xie@eng.ox.ac.uk); [xingcai@mit.edu](mailto:xingcai@mit.edu)

**This PDF file includes:**

Supplementary Text, Fig. S1 and Fig. S2

1. **Automatic single soliton microcomb generation**


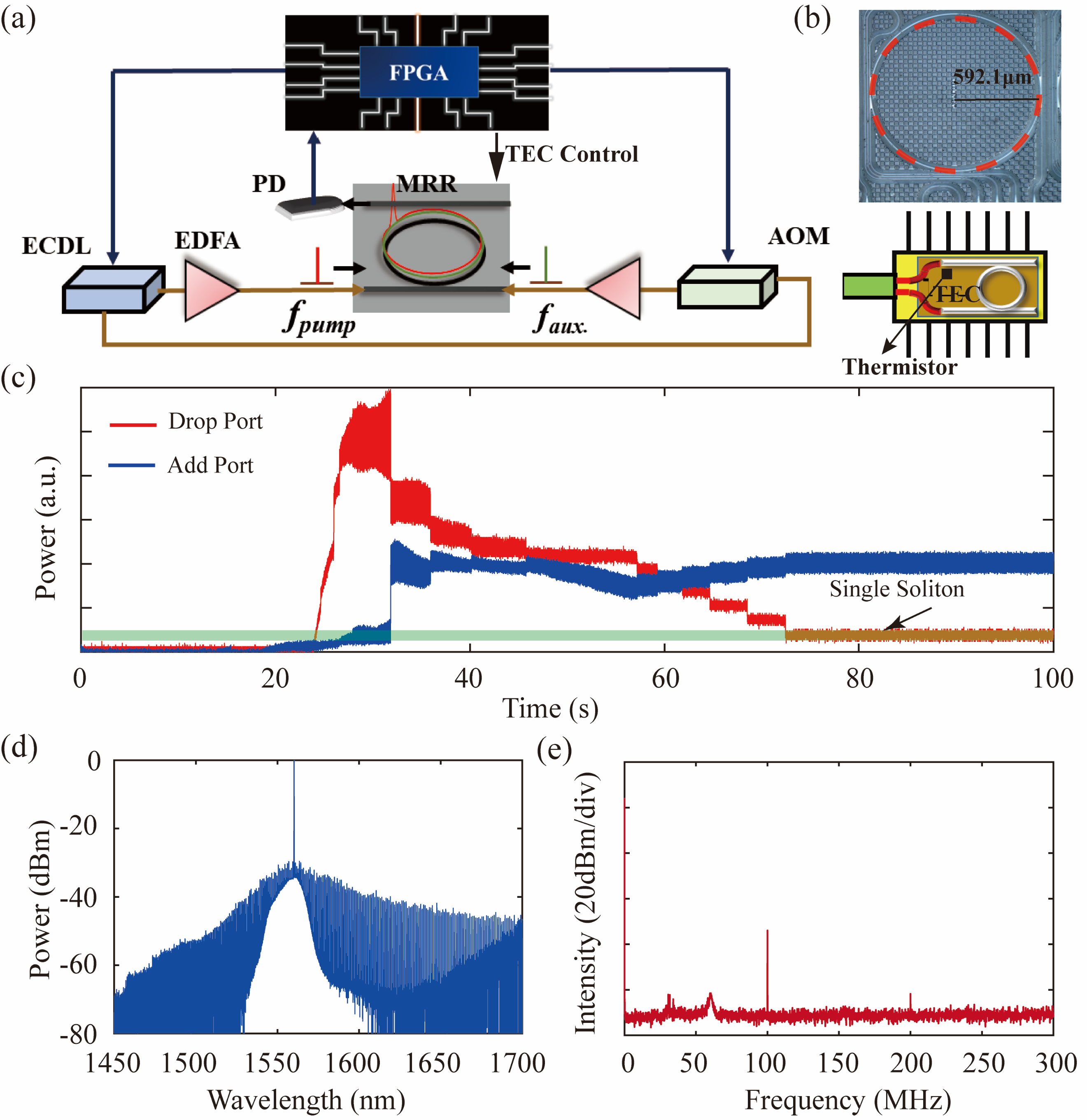


**Fig. S1** **a** Experimental setup of single soliton generation. The auxiliary laser is created via the AOM. The modulation frequency on the AOM and pump laser frequency are controlled via an FPGA system. ECDL: External cavity diode laser, EDFA: Erbium-doped fiber amplifier, AOM: Acousto-optic modulator, PD: Photodetector, MRR: Micro-ring resonator; **b** Microscope image of high-index doped silica glass micro-ring resonator and packaged schematic diagram of MRR. TEC: Thermoelectric cooler; **c** The power traces from the drop port (red line) and add port (blue line). In the red-detuned region, the intracavity power traces for pump-induced microcomb and auxiliary laser demonstrate the opposite trend. The greed line is the single soliton existence power range; **d** Optical spectrum of single soliton microcomb with the repetition rate of ~100 GHz; **e** Beat signal of single soliton microcomb.

In our work, we use an automatic single soliton microcomb as the multi-wavelength light source and carry datasets on different frequency components. Automatic soliton microcomb generation plays an important role in exploring soliton-based applications. Here, we present more details about the experimental setup and results of automatic single soliton microcomb generation in Fig. S1. As is shown in Fig. S1, an automatic single soliton generation system is based on the technique of auxiliary laser heating micro-ring resonator, which is used for maintaining the thermal balance in the soliton existence region [1,2]. Fig. S1 (a) illustrates the experimental setup for the automatic soliton generation. Fig. S1 (b) shows the microscope image of the 49 GHz micro-ring resonator and the schematic diagram of the14-pins butterfly. The frequency of pump laser is shifted via the commercial AOM, which is regarded as the auxiliary laser. By optimizing the modulation frequency on AOM, the single soliton generation is independent of the frequency tuning speed due to the robust thermal balance. The pump laser and auxiliary are amplified to reach the power requirement of soliton formation via commercial polarization-maintaining EFDA and the amplified optical signals are counter-coupled into the micro-resonator. To eliminate the influence of pump laser, the fiber Bragg grating (FBG) is introduced. When monitoring the output power of microcomb from the drop port, the FPGA system could control the pump frequency and the modulation frequency according to the power range of a single soliton microcomb. After optimizing the initial modulation frequency on the AOM to make the microcomb enter into the soliton state, via increasing the pump frequency and modulation frequency, the single soliton is deterministically generated. Fig. S1 (c) shows the power traces from the drop port and add port when the laser gradually enters into the single soliton state from the blue-detuned region to the red-detuned region. The auxiliary laser is heating the cavity when the soliton numbers are gradually decreasing. The single soliton state is completely monitored by the FPGA system. Besides, we also verify the AOM-assisted single soliton generation in other micro-ring resonators. Fig. S1 (d) demonstrates the spectrum of single soliton with the repetition rate of ~100 GHz. During this process, the modulation frequency on AOM is 100 MHz as demonstrated in Fig. S1 (e). Therefore, it demonstrates that the system is stable and suitable for different light source explorations.

1. **Optical coupling and device packaging**

**
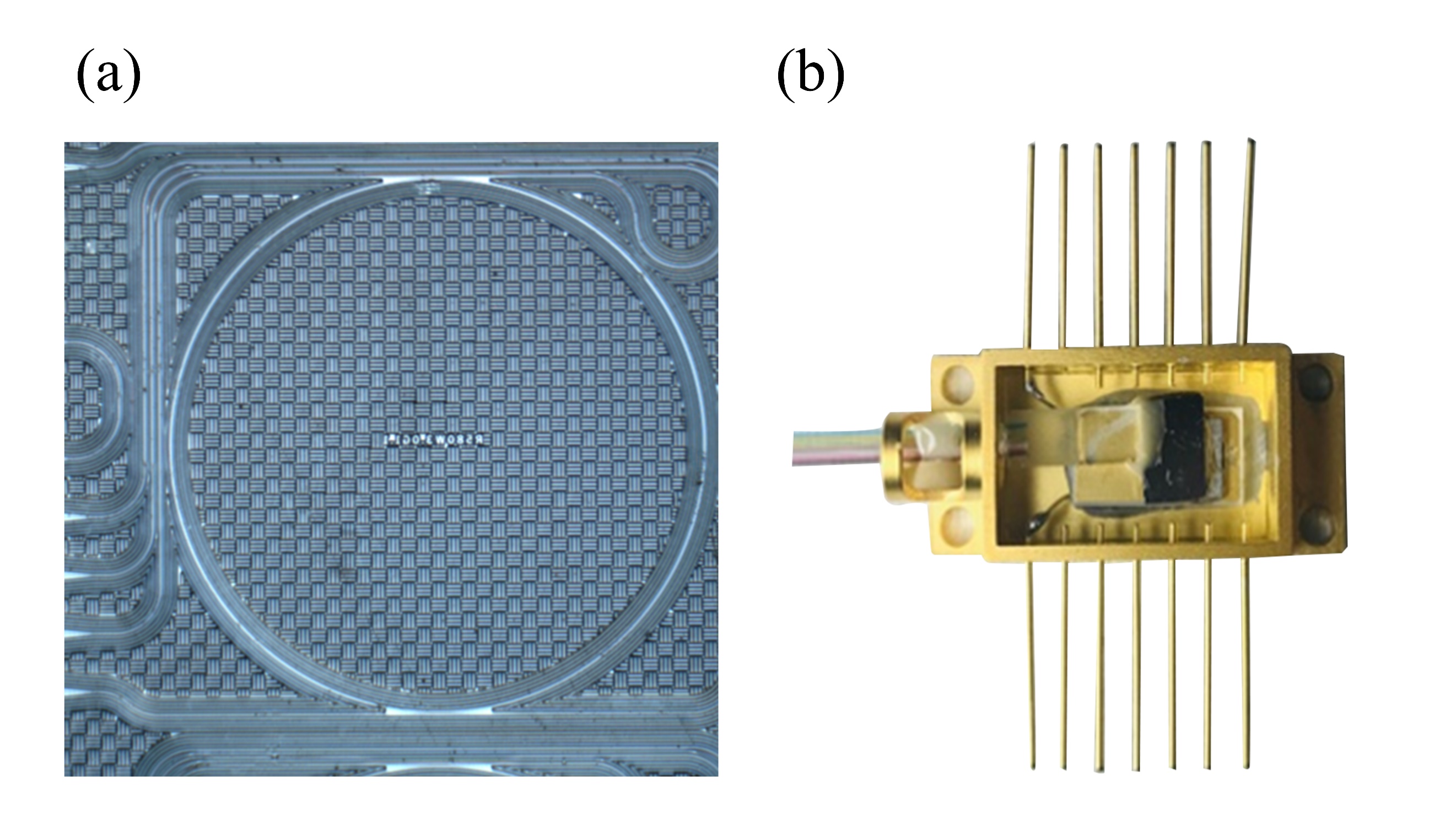
**

**Fig. S2** **a** The microscope image of the micro-ring resonator; **b** The packaged device.

Fig. S2(a) shows the microscope image of the micro-ring resonator based on high-doped index glass. The transmission loss of the waveguide in the near-infrared region is around 0.1 dB/cm and the loss will reduce to 0.05 dB/cm around the communication band. In the practical process of optical coupling between the device and fiber array(FA), the end-face of the chip is polished on a grinding plate by using 1.5 μm abrasive powders mixed with water for 5 minutes. We package the chip with the multi-channel FA on a six-axis coupling stage, which has three linear stages with a resolution of 50 nm and three angle stages with a resolution of 0.003°. Via monitoring the coupling efficiency, the position of FA is adjusted until the inset loss reaches the excepted power value. Furthermore, [index-matching glue](javascript:;) is introduced to glue the chip and FA. Finally, the chip and FA are exposed under an ultraviolet (UV) lamp for 150 seconds and baked in a chamber at 120 °C for 1 hour. In our work, the typical coupling loss is 1 to 2dB per facet. After that, we furtherly package the chip in a butterfly shell, as is shown in Fig. S2(b). We conglutinate a TEC chip of the geometrical size with 10.2 mm x 6.05 mm to the baseplate of a butterfly package using silver glue and then solder the two electrodes of the TEC chip to two pins of the butterfly package, which would contribute to controlling the temperature. Next, we paste a tungsten plate with 5 mm × 5 mm × 1 mm to the surface of the TEC chip, which is used for filling the gap between the TEC and chip as a heat sink. And paste the micro-ring resonator chip to the top of the tungsten plate and fix the pigtail of the FA to the output port of the butterfly package. Besides, paste a thermistor chip to the surface of the TEC chip and connect one electrode of the thermistor to the top surface of the TEC chip. Furtherly, the wire bonds the other electrode of the thermistor and the top surface of the TEC chip to the pins of the butterfly package. Finally, the packaged device is baked at 100 °C for 1 hour to solidify the silver glue.

[S1] X. Wang, P. Xie, W. Wang, Y. Wang, Z. Lu, et al., Program-controlled single soliton microcomb source. Photonics Res. **9**, 66 (2021). https://doi.org/10.1364/PRJ.408612

[S2] R. Miao, C. Zhang, X. Zheng, X. Cheng, K. Yin, et al., Repetition rate locked single-soliton microcomb generation via rapid frequency sweep and sideband thermal compensation. Photonics Res. **10**, 1859 (2022). https://doi.org/10.1364/PRJ.458472
